# mTOR: An attractive therapeutic target for osteosarcoma?

**DOI:** 10.18632/oncotarget.9305

**Published:** 2016-05-11

**Authors:** Liu Ding, Liu Congwei, Qing Bei, Yang Tao, Wang Ruiguo, Yu Heze, Dou Bo, Li Zhihong

**Affiliations:** ^1^ Department of Orthopaedics, The Second Xiangya Hospital of Central South University, Changsha, Hunan, China; ^2^ Department of Thoracic Surgery, The Second Xiangya Hospital of Central South University, Changsha, Hunan, China; ^3^ Department of Otolaryngology, Head and Neck Surgery, The Second Xiangya Hospital of Central South University, Changsha, Hunan, China; ^4^ Clinical Medicine for Eight-Year-Program, Xiangya School of Medicine, Central South University, Changsha, Hunan, China

**Keywords:** osteosarcoma, mTOR, target, autophagy, apoptosis

## Abstract

Osteosarcoma (OS) is a common primary malignant bone tumor with high morbidity and mortality in children and young adults. How to improve poor prognosis of OS due to resistance to chemotherapy remains a challenge. Recently, growing findings show activation of mammalian target of rapamycin (mTOR), is associated with OS cell growth, proliferation, metastasis. Targeting mTOR may be a promising therapeutic approach for treating OS. This review summarizes the roles of mTOR pathway in OS and present research status of mTOR inhibitors in the context of OS. In addition, we have attempted to discuss how to design a better treatment project for OS by combining mTOR inhibitor with other drugs.

## INTRODUCTION

Osteosarcoma (OS) is the most common primary bone malignant neoplasm in children and young adults which is featured with high local aggressiveness and distant organic metastasize [1]. Despite great advances in treatments, comprising neoadjuvant chemotherapy and surgical technology, a notable number of relapse or metastasis still occur [2, 3]. The cure rate of OS is approximately 25 % when accompanied with metastasis at the time of diagnosis, which remains almost stagnant over the past 20 years [4, 5]. Thus, novel chemotherapy drugs are urgently needed.

Mammalian target of rapamycin (mTOR), a downstream mediator in the phosphatidylinositol 3-kinase(PI3K) signaling pathway, is an essential serine/threonine kinase [6]. It involves in regulating important cellular functions including survival, cell growth, proliferation, migration and angiogenesis [7, 8]. Recently, growing researches show aberrant activation of mTOR in many cancer including human osteosarcoma [9]. Notably, the inhibitors of mTOR can demonstrate anti-tumor effect in OS by inhibiting cell growth and proliferation, which raises great interesting in exploring available drug targeting mTOR to improve survival rate of OS [8].

In this review, the role of mTOR pathway and present inhibitors targeting on mTOR in OS are summarized. In addition, we also discuss the strategy reversing resistance to chemotherapeutics for OS patients.

## OVERVIEW OF THE MTOR PATHWAY

mTOR is a serine/threonine kinase, which acts as a central controller in regulating important cellular functions [6]. It exists in two multiprotein complexes, mTOR complex 1(mTORC1) and mTOR complex 2(mTORC2). mTORC1 consists of mTOR, regulatory associated protein of mTOR (Raptor), mLST8(mammalian lethal with SEC13 protein 8)/G-protein α-subunit like protein (GαL), RAS40 and Deptor [10]. While mTORC2 is composed of rapamycin-insensitive companion of mTOR (Rictor), mTOR, mLST8/GαL, proline-rich repeat protein-5 (PRR-5)/protein observed with Rictor-1 (Protor-1), stress-activated-protein-kinase-interacting protein 1 (Sin1), and Deptor [11]. Despite both mTORC1 and mTORC2 can be restrained by rapamycin, mTORC1 seem to be relatively sensitive to it [12].

The main upstream signals of mTORC1 are adenosine 5′-monophosphate (AMP)-activated protein kinase (AMPK) and PI3K pathway [13, 14]. PI3Ks constitute a lipid kinase family. Once activated, its catalytic subunit activates AKT. Subsequently mTORC1 is activated. Another upstream effector, AMPK, is a key energy sensor [15], which can regulate cellular metabolism. Activation of AMPK by nutrient deprivation promotes mTORC1 inactivation. The downstream mediators of mTORC1 include ribosomal S6 protein kinase 1 (S6K1) and eIF4E-binding protein 1 (4E-BP1), cyclin dependent kinases (CDKs) and the hypoxia-inducible factor 1α (HIF1α), which promote the expression of a wide range of glycolytic genes [16]. Thus, in the nutrient rich environment, mTORC1 is stimulated and promotes protein synthesis, cellular growth as well as the inhibition of autophagy, a saving program to survive starvation [17].

**Figure 1:**
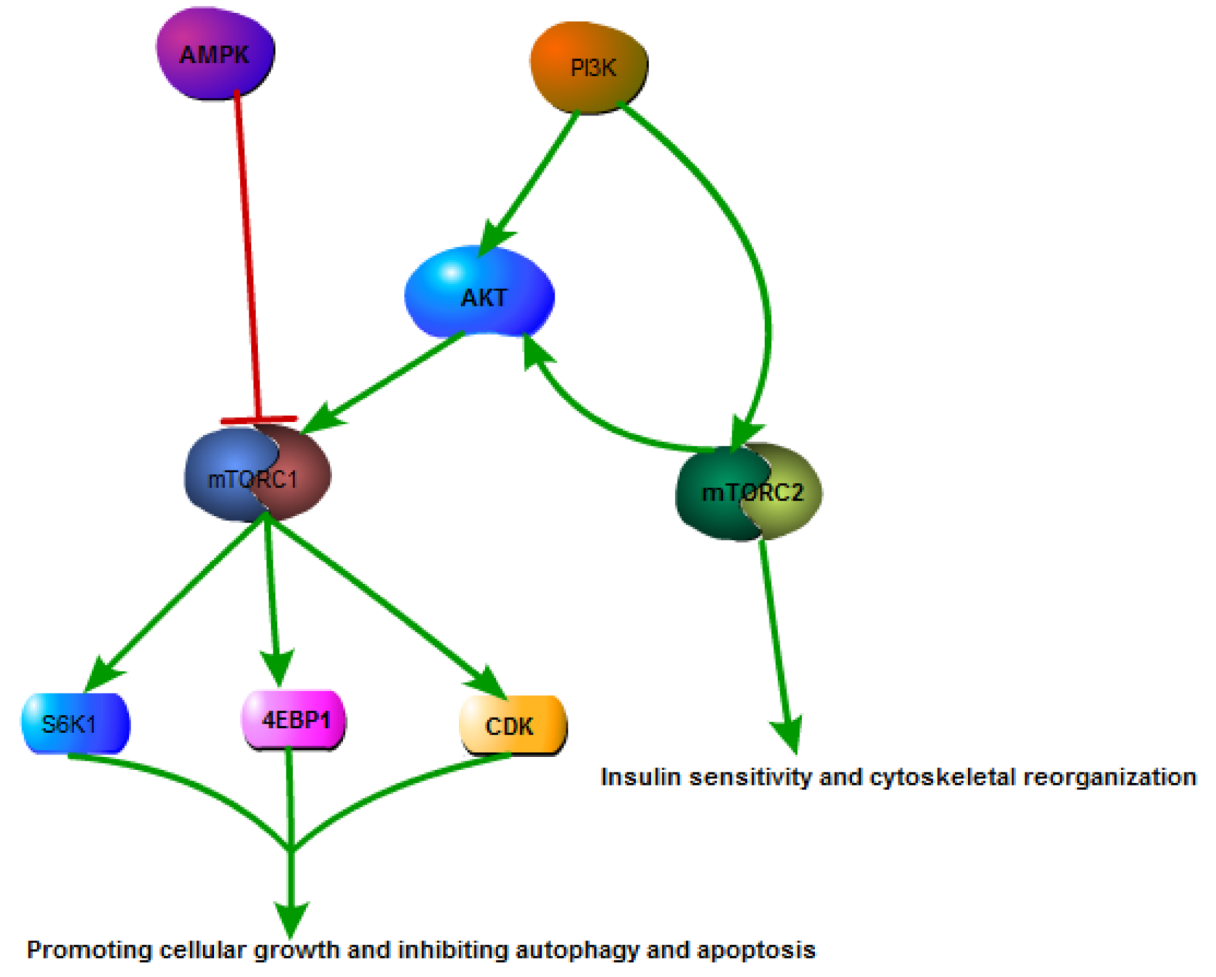
Overview of mTOR signaling pathway Activation of PI3K/AKT pathway can stimulate mTORC1, meanwhile mTORC1are negatively regulated by AMPK. Activation of mTORC1 upregulates CDK and phosphorylates S6K1 and 4EBP1, modulating cellular growth, autophagy, and apoptosis process. Additionally, PI3K is also the upstream controller of mTORC2, activation of which phosphorylates AKT, forming a positive feedback to enhance the signal. Moreover, activation of mTOR2 is involved in insulin sensitivity and cytoskeletal reorganization.

Compared with mTORC1, the upstream pathways of mTORC2 are less known. PI3K is regarded as a direct upstream effector of mTORC2 [18], while AKT is the main target. Stimulation of AKT by mTORC2 activates mTORC1, thus forming a positive feedback to enhance the signal (Figure 1). Besides, mTORC2 is related to insulin sensitivity and cytoskeletal reorganization [19, 20].

## ROLES OF THE MTOR PATHWAY IN OS

### Promoting cellular growth and proliferation

Activation of mTOR pathway is a important signaling pathway stimulating cell growth and proliferation [17]. Aberrant activation of mTOR has been detected in OS [9]. Rapamycin is a common mTOR inhibitor. Treatment with rapamycin suppressing OS cell growth and proliferation has been well documented [21]. Moreover, Rapamycin can effectively inhibit osteosarcoma stem cells proliferation [22]. Additionally, some moleculars and drugs, such as lupeol, Oleanolic acid, metformin, p53, icariside II, capsaicin, phosphorus-containing sirolimus, heat shock protein 90B1, inhibit OS cell growth and proliferation by targeting AMPK/mTOR and PI3K/AKT/mTOR signaling and down-regulating cyclin D1 and phosphorylation of S6K1 and 4EBP1, which are regarded as downstream target of mTORC1 [23-30]. Besides, overexpression of miR-101 can down-regulate the expression of mTOR, contributing to the inhibition of OS cell proliferation [31]. Moreover, activation of PI3K/mTOR signaling by X-Box Binding Protein 1 correlates to Poor Prognosis [32]. Taken together, mTOR play a vital role in promoting growth and proliferation in OS.

### Inducing cellular metastasis

Distant organic metastasize remains the predominant lethal for cancer patients. Thus, how to prevent metastas presents a great challenge. It has been proved that mTOR has potential function on facilitating metastasis. Notablely, rapamycin reduces tumor cell metastasis in a murine model of osteosarcoma *via* blocking the mTOR/S6K1/4E-BP1 pathway [33, 34]. Metformin exerts markedly anti-metastatic potentials by downregulating matrix metalloproteinases, which have an ability of degrading extracellular matrix to facilitate tumor cell metastasis [25, 35-36]. In addition, the histone deacetylase inhibitor and P53 can also downregulate mTOR to restrain metastasis [26, 37]. Another pathway by which activation of mTOR pathway promotes OS cell metastasis is angiogenesis. P53 and phosphorus-containing sirolimus suppresses OS cell angiogenesis through inhibition of mTOR [26, 30]. Thus, inhibition of mTOR may be a novel effective candidate therapeutic strategy against OS cell metastasis.

### Inhibition of apoptosis

Apoptosis is refered to a process of programmed cell death which occurs in multicellular organisms [38, 39]. Chemotherapy kills cancer cell mainly by inducing apoptosis. Therefore, developing an effective proapoptotic drug seemed to be a good therapeutic candidate for OS. Interestingly, many findings demonstrate that inhibition of mTOR pathway can induce apoptosis of OS cell [26, 27, 29, 31, 40-44]. At the same time, α-Elemene, isolated from herbs and plants, upregulates HIF-1αprotein *via* PI3K/Akt/mTor signaling pathway, contributing to inhibition of apoptosis [45]. Moreover, overexpression of miR-101 can suppress the expression of mTOR, inducing the apoptosis of OS cell [31]. Therefore, drug suppressing mTOR pathway has pro-apoptotic effect, which may be a useful therapeutic option for OS.

### Suppression of autophagy

**Figure 2:**
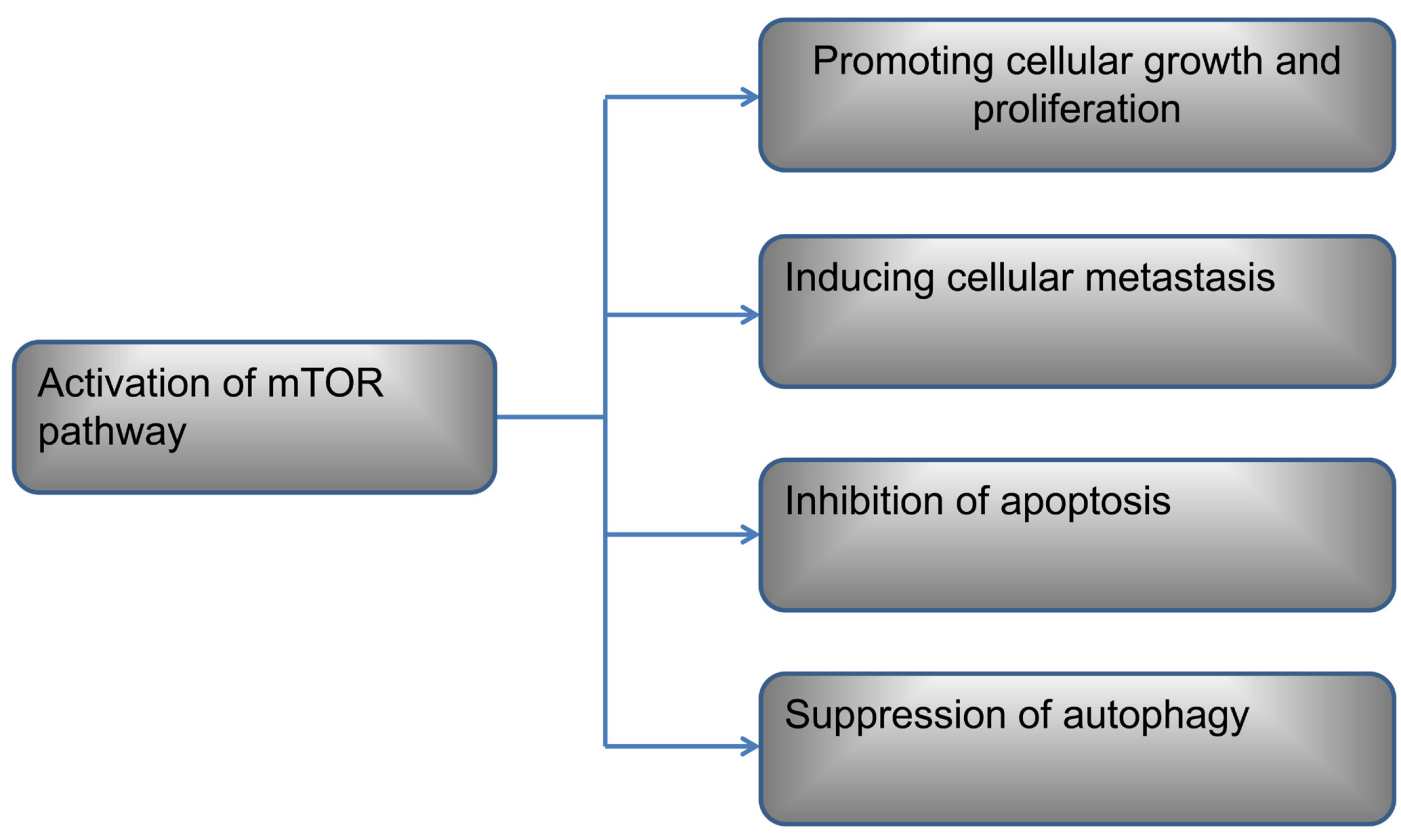
The roles of mTOR pathway in OS cell

Autophagy is a cellular physiological process which delivers cytoplasmic material to the lysosome to provide energy and nutrients [46, 47]. It occurs as a strategic survival mechanism that reuses energy and nutrients under special conditions [48, 49]. Thus, autophagy is regarded as an emergency pathway of protecting cells from adverse microenvironment. Surprisedly, autophagy is also detected in OS cell [50]. Inhibition of mTOR in OS cell leads to autophagy which has advantage effect on cell [51-52]. Meanwhile, inhibition of autophagy has a negative impact on osteosarcoma tumors [50]. Therefore, activation of mTOR induces autophagy, which is regared as a prosurvival response contributing to drug resistance. Moreover, treating with autophagy inhibitors may lead OS cell apotosis [53]. Nevertheless, activation of autophagy by rapamycin also leads to OS cell death. This mechanism may be due to the extent of autophagy activation beyond the reversibility of cell viability, contributing to out of control of autophagy process [54-58]. Taken together, the signaling pathways involved in autophagy are still little known. In addition, in view of the mTOR is the mutual upstream controller of apoptosis and autophagy process, breaking the balance between apoptosis and autophagy and shifting to apotosis after activation of mTOR pathway may be a promising strategy for facing the challenges of OS. Further investigations are needed to help us understand completely about the roles of mTOR pathway in OS (Figure 2).

## INHIBITORS OF MTOR

Despite great advances in treating OS, significant improvement in survival rate and survival time is not acquired. The reason is that cancer cell exerts resistance to chemotherapy drug in clinical application, even it shows promising anti-tumor activity in pre-clinical test. Surprisingly, overactivation of mTOR pathway may relate to resistance to chemotherapy drug [59]. Therefore, the combination of chemotherapy drugs and mTOR inhibitors may demonstrate synergistic effects. Consistent with this notion, C6 ceramide can sensitize pemetrexed-induced apoptosis and cytotoxicity *via* inactivation of AKT-mTOR signaling in OS [59]. Moreover, specil inhibition of mTORC2 but not mTORC1 can promote cisplatin-induced apoptosis [60]. Thus, exploring novel mTOR inhibitors raise great interest treating OS.

Table 1 lists present research status of mTOR in the context of OS.

**Table 1:** Research status of mTOR in the context of OS

Publication	Name	Main Findings	Ref
2005,2009 2013,2015	Rapamycin	Rapamycin can inhibit OS cell proliferation, metastasis, and induce autophagy.	[22][33][34] [44][61]
2010	Everolimus	Combination with ZOL(zoledronate, an anti-osteoporotic drug) augments the inhibition of Everolimus in cell proliferation.	[65]
2011	Oleanolic acid (OA)	OA exhibits potent anti-tumor activity against osteosarcoma cells	[23]
2011	Cucurbitacin B	Cucurbitacin B alone or in combination with methotrexate(MTX) exerts anti-tumor effects on human OS	[66]
2012	Ridaforolimus	In Phase II study, ridaforolimus shows promising anti-proliferative activity against OS	[62]
2013	Everolimus	Sorafenib in combined with everolimus contributes to an increasing antitumor activity	[67]
2014	NVP-BEZ235	NVP-BEZ235, a dual PI3K/mTOR inhibitor, shows promising antitumor activity in OS.	[72]
2014	Temsirolimus	Temsirolimus combined with cisplatin or bevacizumab exerts synergistic effects for treatment of OS.	[68]
2014	PP242	Inhibition of mTORC2 effectively promotes cisplatin-induced apoptosis	[60]
2014	Temsirolimus, LY294.002 and PP242	mTOR inhibitors can blunt the p53 response to nucleolar stress in OS.	[79]
2015	Rapamycin	JQ1 and rapamycin synergistically inhibite the growthl of OS cells in vitro and in vivo.	[69]
2015	Temsirolimus	In this phase II trial the combination of cixutumumab and temsirolimus does not show objective result.	[78]
2015	Everolimus	The combination of sorafenib and everolimusdoes not attain the prespecified target of 6 month PFS in a non-randomised phase 2 clinical trial	[70]
2015	MLN0128	MLN0128 exerts anti-tumor activity in in vitro and in vivo model of OS.	[63]
2015	NVP-BEZ235	NVP-BEZ235 shows promising anti-tumor activity, which is enhanced by MEK/Erk inhibitors	[73]
2015	INK-128	INK-128 exibit potent anti-OS activity in vitro and in vivo.	[64]
2016	Rapamycin	The combination of rapamycin and an autophagy inhibitor exerts synergistic effects for treatment of OS byeffectively promoting the apoptotic pathway.	[71]

mTOR inhibitor suppresses OS cell growth solely *in vivo* and *in vitro* and phase II study [22, 23, 33, 34, 44, 61-64]. Besides, Some reports find mTOR inhibitor achieves an increasing anti-tumor effect when combining with other forms of drugs, such as anti-osteoporotic drug, extra terminal domain protein inhibitor, conventional chemotherapy drugs [65-71]. In addition, a dual PI3K/mTOR inhibitor shows an promising result in treating OS cell, and this anti-tumor activity can be enhanced by MEK/Erk inhibitors [72, 73].

The roles of autophagy in OS cell survival and death are paradoxical and complex [74] just as we talk above. Notablely, some researchers pay attention to inhibiting both mTOR and antophagy process for treating OS. Heat shock protein 90 (Hsp90), an abundant molecular chaperone, is involved in cell growth, differentiation and survival [75, 76]. Hsp90 inhibitor suppresses mTOR, contributing to autophagy. However, in combination with antophagy inhibitor, hsp90 exerts a much greater extent apoptosis [77]. Another finding also shows that rapamycin induces the apoptosis of OS cells, which is enhanced by antophagy inhibitor [71]. Thus, treating OS cell with mTOR inhibitor alone may inhibit the proliferation and promotion of OS cell by targeting mTOR pathaway. However, as the ability of pro-apoptosis is growing, the escape pathway of autophagy is triggered, counteracting the anti-tumor effect of mTOR inhibitor and contributing resistence to mTOR inhibitor, which is consistent with the modest anti-tumor effect of mTOR inhibitor in clinical application. Autophagy inhibitor can elevate efficiency of mTOR inhibitor by blocking autophagy process in treating OS. Owing to partly understand in the autophagy pathway in OS, further investigations are needed.

Overall, mTOR inhibitor combined with other drugs may provide a novel therapeutic strategy against OS. However, the combination of the anti-insulin-growth factor type 1 receptor antibody and mTOR inhibitor does not show a objective result in an phase II trial [78]. The different conditions of cell living in between pre-clincal test and clinical study and the distrinct type of drug combined with mTOR inhibitor may lead to dissatisfied result. Moreover, nucleolar stress, induced by chemotherapeutic drugs, stimulates p53-dependent signaling pathways which contribute to cell cycle arrest, apoptosis, and mTOR inhibitor can alleviate this p53 response to nucleolar stress [79-85]. The cross-linking of p53-dependent signaling pathways and mTOR pathway may explain this inconsistent result. Thus, we should take the complexity and potential problems into consideration when mTOR inhibitor combined with other cytotoxic compounds is applied in treating OS.

Taken together, the combination of mTOR inhibitor and other drugs may provide an efficient therapeutic strategy against OS. However, the mTOR signaling pathway is complexity in OS, and its roles in OS are still not completely understood. Further studies will help us design a combinatorial chemotherapy regimen against OS.

## CONCLUSIONS

Activation of mTOR pathway promotes OS cell proliferation, metastasis, and inhibits the intracellular processes of apoptosis and autophagy. mTOR inhibitor used alone exerts a promising anti-tumor activity, which is enhenced by combining with other drugs for OS. Thus, exploring a better combinatorial chemotherapy regimen provide a novel therapeutic approach for OS. However, the detail mechanism of mTOR pathway and synergistical effect of mTOR inhibitor and other drugs in OS are still not fully understood. Therefore, future further researches are required to gain a better understanding.
